# Supplementary material for: Metaepigenomic analysis reveals the unexplored diversity of DNA methylation in an environmental prokaryotic community
Source: Nat Commun. 2019 Jan 11;10:159. doi: 10.1038/s41467-018-08103-y (PMC6329791; doi:10.1038/s41467-018-08103-y)
Supplement: Supplementary file 1 — Supplementary Information [file 41467_2018_8103_MOESM1_ESM.pdf]

SUPPLEMENTARY INFORMATION

Metaepigenomic analysis reveals the unexplored diversity of DNA methylations in an environmental prokaryotic community.

Satoshi Hiraoka[1, †, *], Yusuke Okazaki[2], Mizue Anda[3], Atsushi Toyoda[4], Shin-ichi Nakano[2], Wataru Iwasaki[1,3,5,*]

*Correspondence should be addressed to:
hiraokas@jamstec.go.jp
Iwasaki@bs.s.u-tokyo.ac.jp

This PDF file includes:
Supplementary Figures 1 to 7.
Supplementary Tables 1 to 4.

SUPPLEMENTARY FIGURES

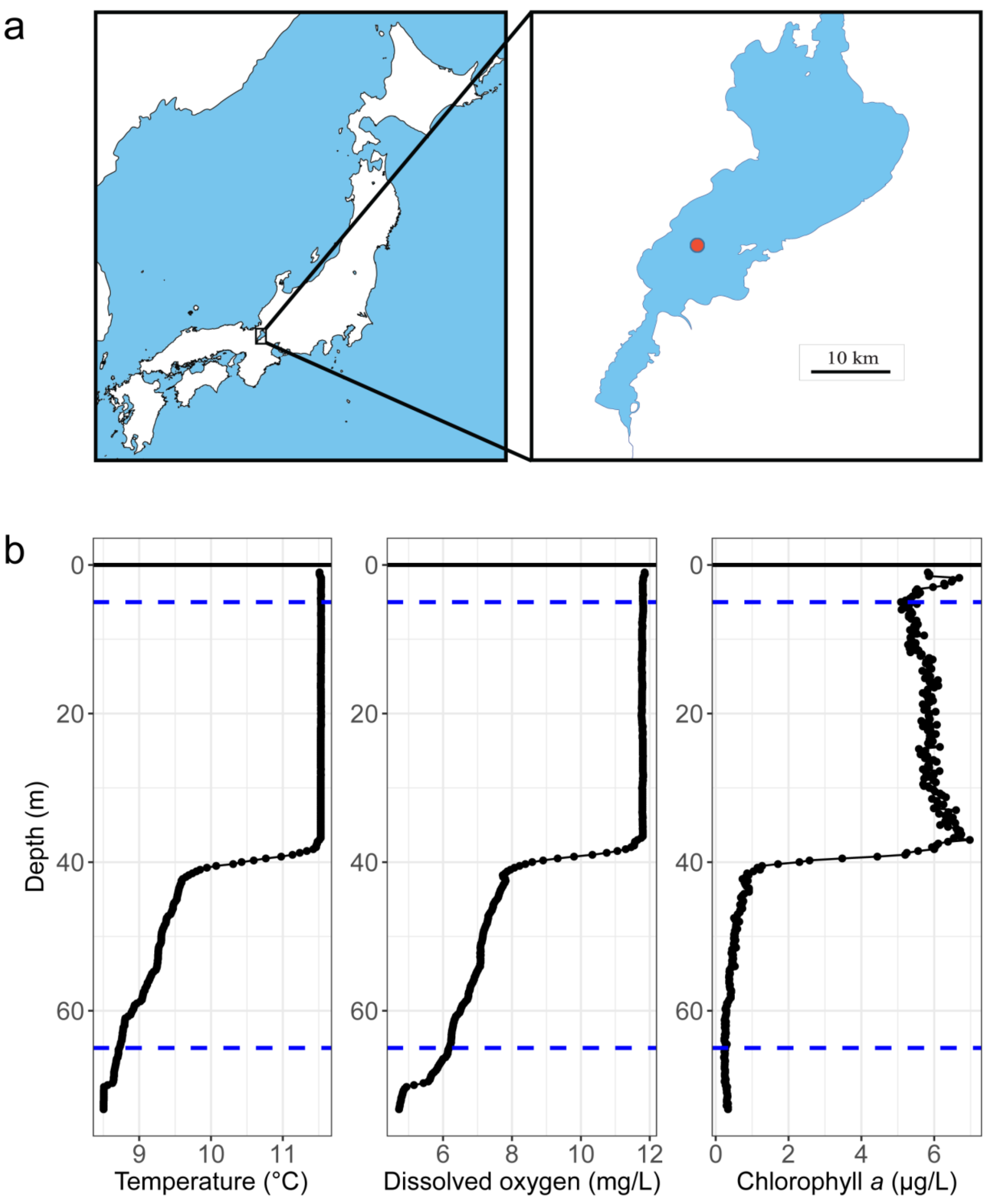

Supplementary Figure 1. Sampling site characteristics. **a** Map of the Japanese archipelago (left) and Lake Biwa (right). The red point indicates the sampling site. The map data were retrieved from d-maps.com (https://d-maps.com/carte.php?num_car=346 and https://d-maps.com/carte.php?num_car=12536, respectively). **b** Vertical profiles (water temperature, dissolved oxygen concentrations, and chlorophyll *a* concentrations) measured at the sampling site. The two blue dashed lines represent the sampling depths (*i.e.*, 5 m and 65 m).

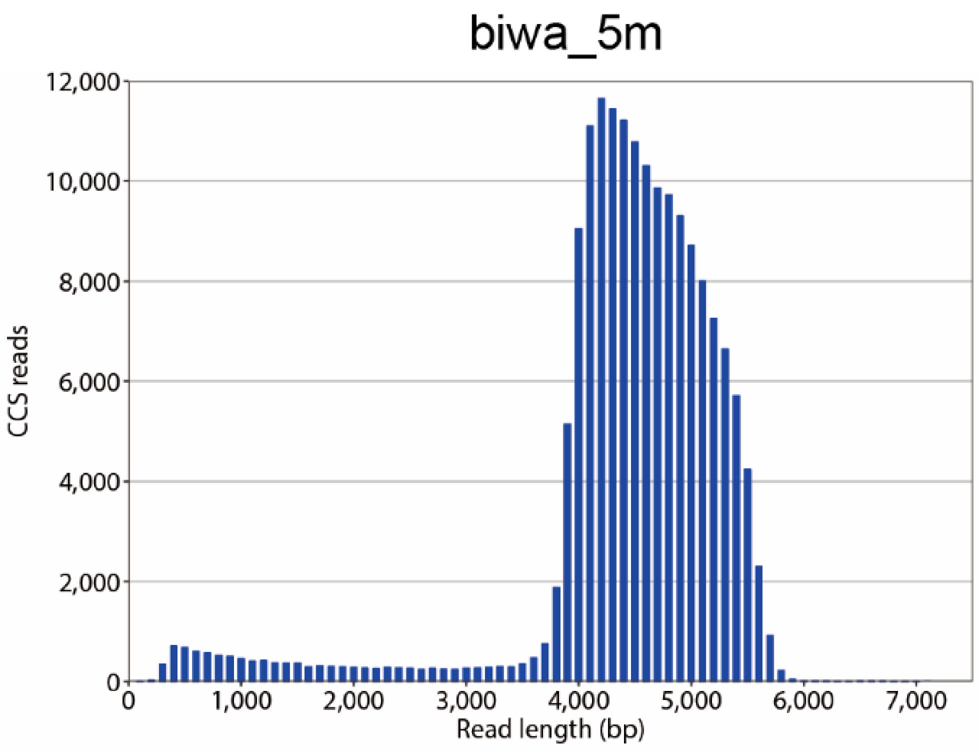

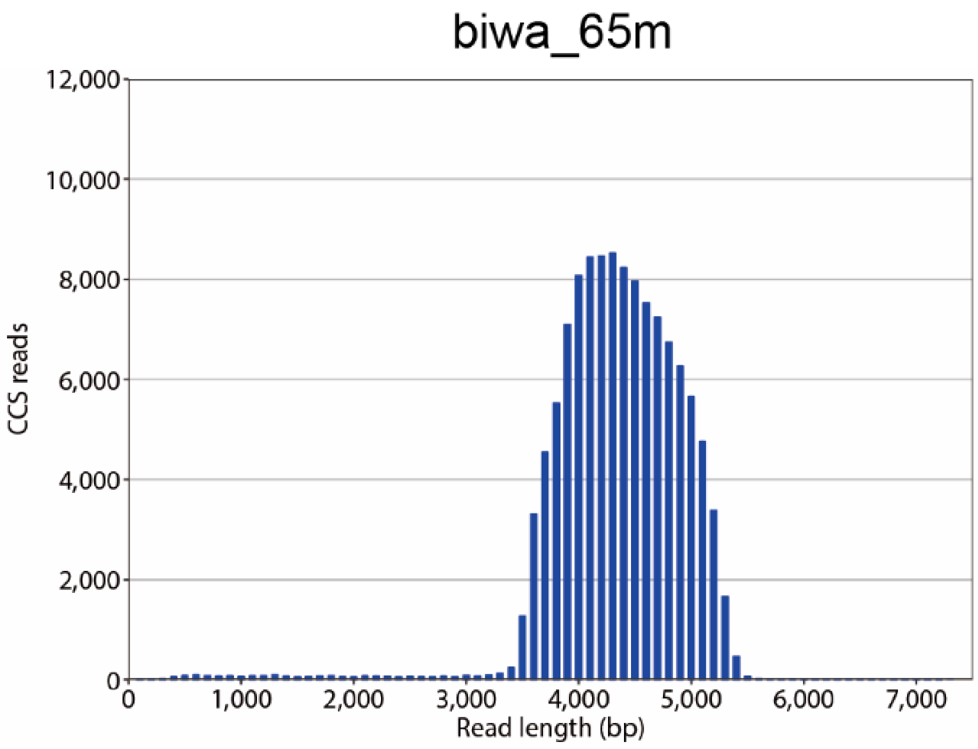

Supplementary Figure 2. Frequency distribution of the length of the circular consensus sequence (CCS) reads. The SMRT libraries were size-selected at a 4-kbp length. The read lengths were binned in 100-bp increments.

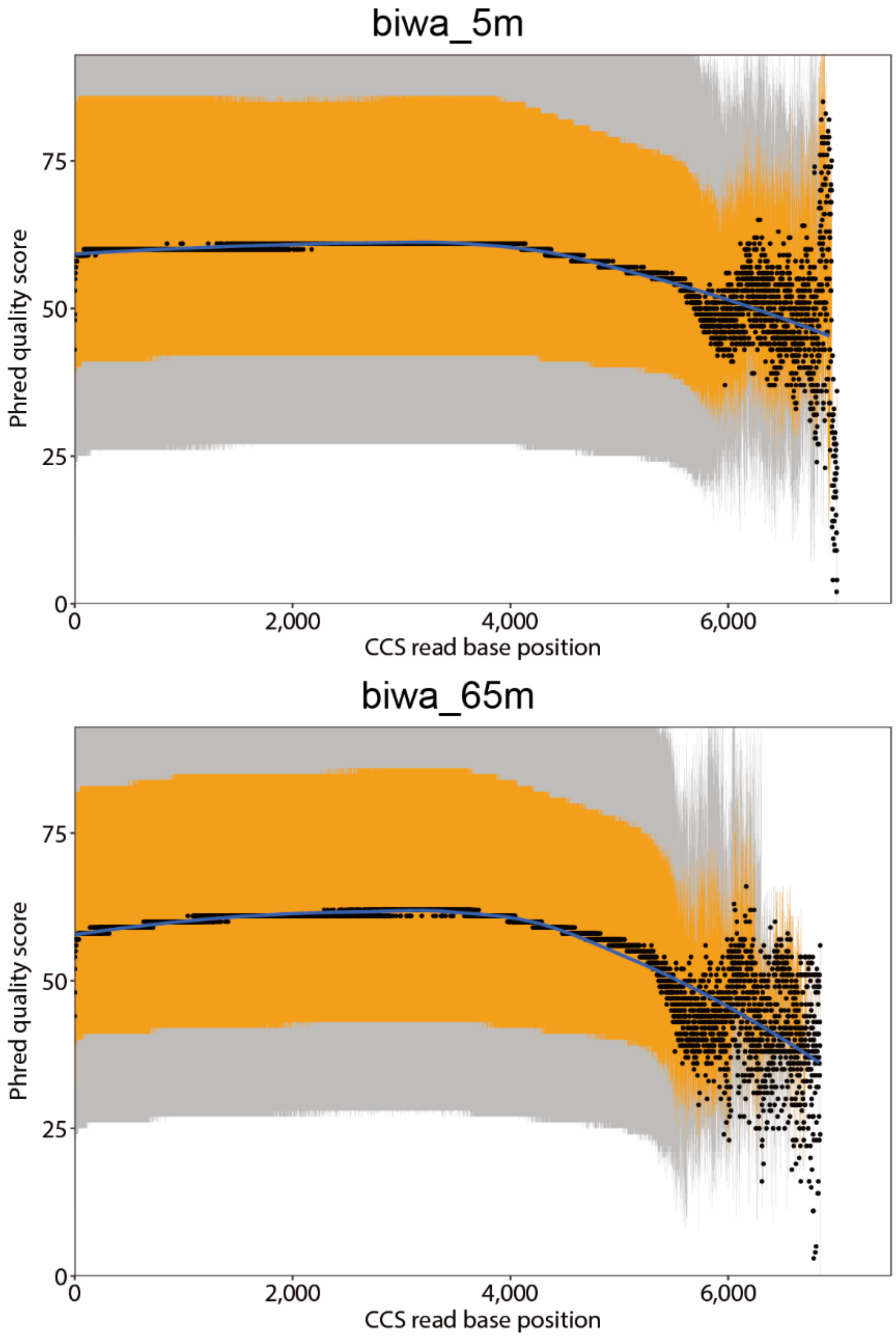

Supplementary Figure 3. Base quality scores of the CCS reads. Gray areas represent the 10th to 90th percentiles of Phred quality scores at each base position. Orange areas represent the 25th to 75th percentiles. Dots represent mean values. Blue lines show the fitted LOESS curves.

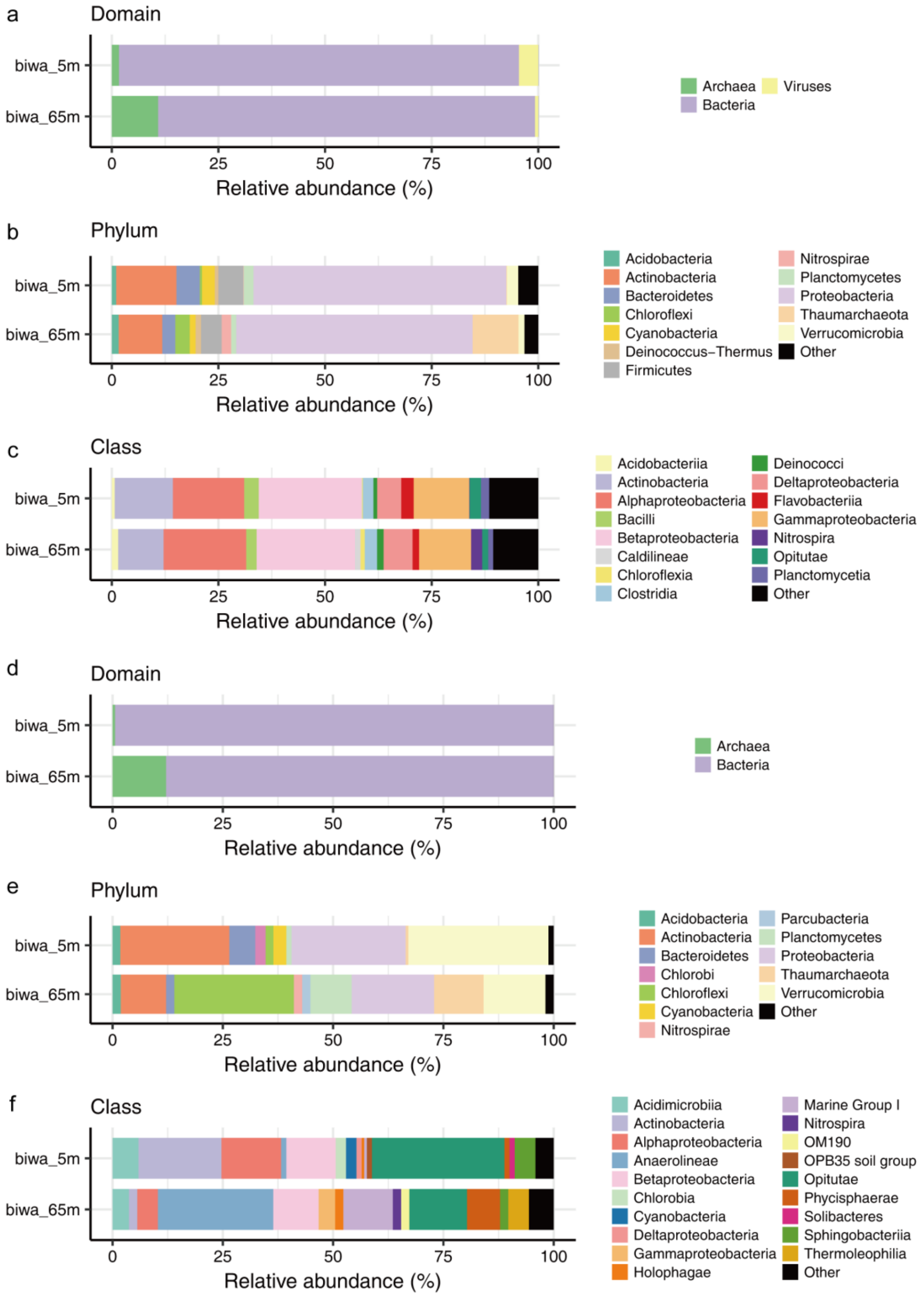

Supplementary Figure 4. Phylogenetic distribution of CCS reads. Estimated relative abundances obtained using Kraken with RefSeq complete prokaryotic and viral genomes at the (**a**) domain, (**b**) phylum, and (**c**) class levels and estimates from BLAST analysis of 16S rRNA sequences with the SILVA database at the (**d**) domain, (**e**) phylum, and (**f**) class levels are shown. The eukaryotic and viral reads are ignored, and groups showing <1% abundance are grouped as 'Others' in **b**, **c**, **e**, and **f**.

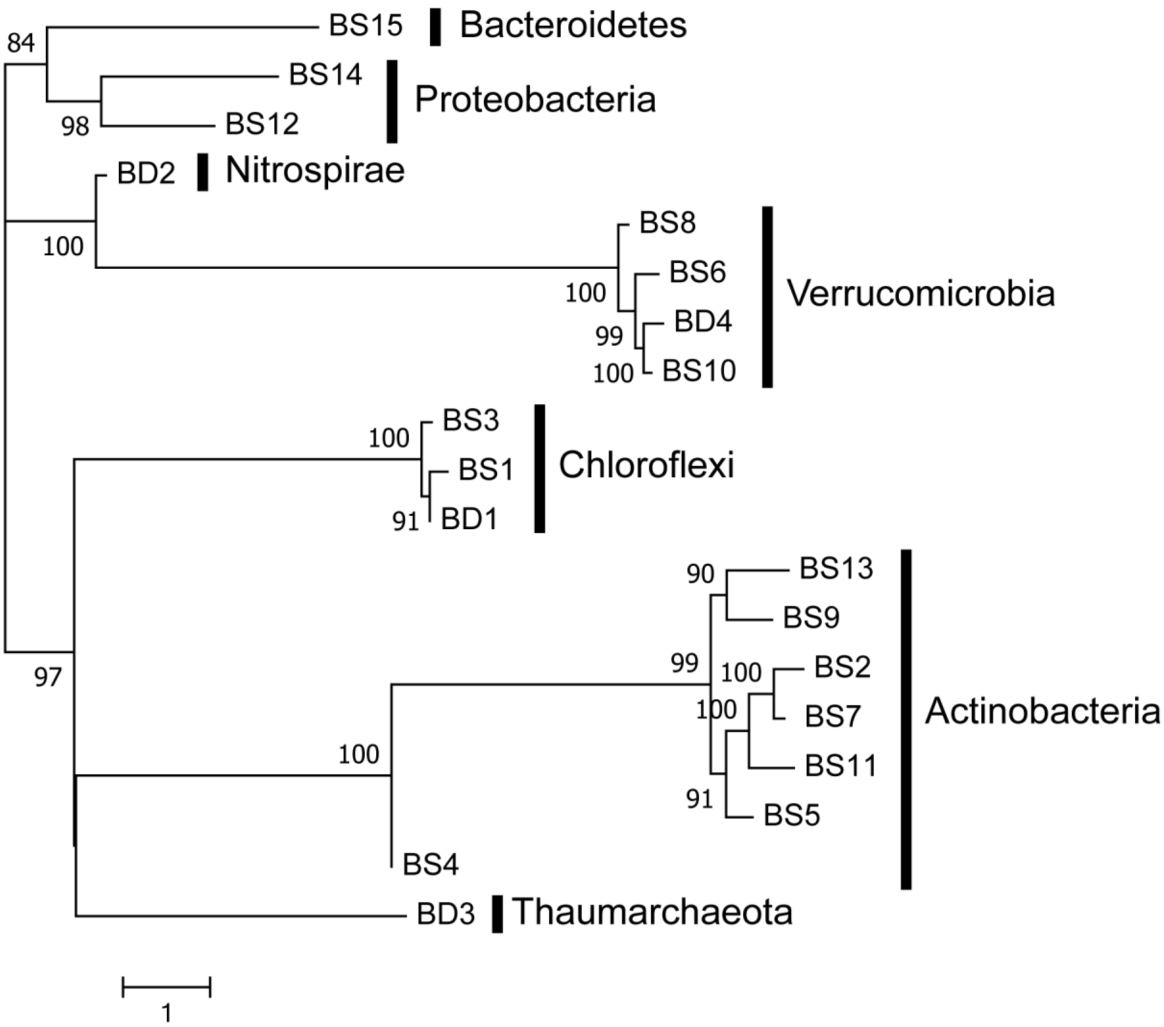

Supplementary Figure 5. Phylogenetic tree of draft genomes. The phylogenetic tree was reconstructed using a set of up to 400 conserved bacterial marker genes with 1,000 bootstrap replicates via the maximum-likelihood method. Support values greater than 80% are shown.

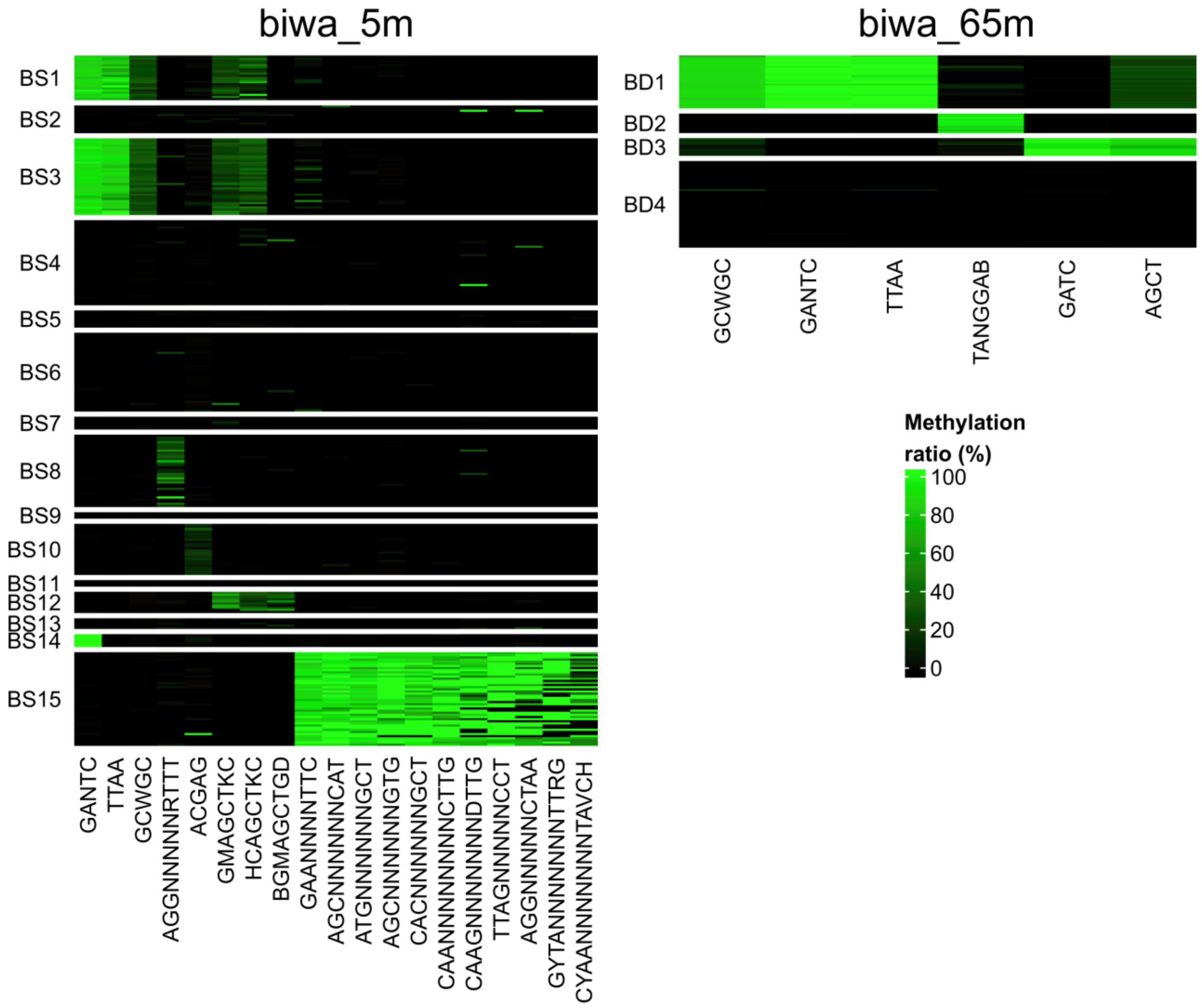

Supplementary Figure 6. Ratios of methylated motifs on each contig. The green color represents the methylation ratios of all motifs detected in this study. If a motif sequence is absent from any contig, the ratio was regarded as zero. While contigs in each draft genome showed a similar methylation pattern in general, several contigs showed methylation patterns that seem to be inconsistent to those of the other contigs in the same genome bin. These inconsistencies could be due to errors in the genome binning and be used for improving the assembly and binning; however, there was no case that the inconsistency was concluded to be due to a binning error instead of a modification detection error. For instance, the ~10 kbp contig of the BS15 genome on which methylated ACG**A**G was detected contained only two ACGAG sequences, that is, two modification detection errors could also explain the observed inconsistency. In addition, the global detection of low-level methylation of GMAG**C**TKC and HCAG**C**TKC motifs in the BS1 contigs and AG**C**T in the BD1 contigs were likely due to methylation of the overlapping G**C**WGC motif.

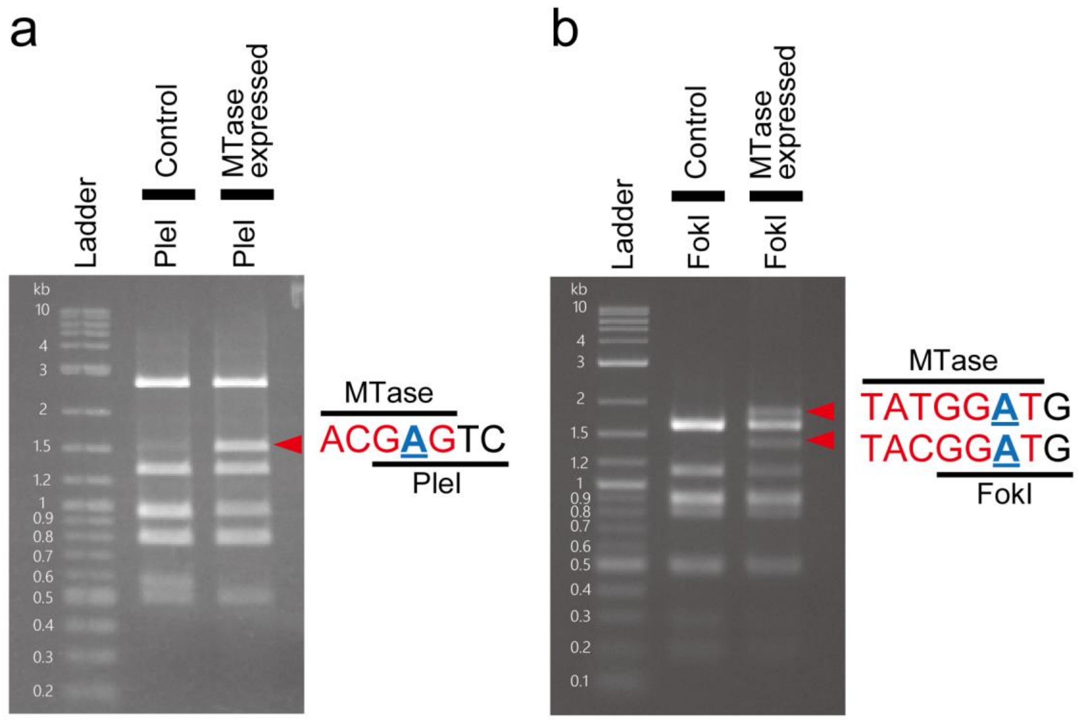

Supplementary Figure 7. REase digestion assays. **a** Assay of the EMGBS10_10070 gene. PleI was employed for the assay, where the plasmid contained 10 GAGTC target sites. Based on the results of the metaepigenomic analysis, ACG**A**G was hypothesized to be the true methylation motif of the EMGBS10_10070 protein. There is one site where the PleI target site overlaps with the hypothesized motif in the plasmid (schematically represented at right), and failure of cleavage at that site was computationally predicted to produce a 1.5-kb fragment. When EMGBS10_10070 was expressed in the cell, such a fragment was actually observed (indicated by a red mark). This result supports the hypothesis that the methylation motif of EMGBS10_10070 is ACG**A**G. An NEB 2-log DNA ladder was used as a size marker. **b** Assay of the EMGBD2_08790 gene. FokI was employed for the assay, where the plasmid contained 14 GGATG target sites. Based on the results of the metaepigenomic analysis, TANGG**A**B was hypothesized to be the true methylation motif of the EMGBD2_08790 protein. There are two sites where the FokI target sites overlap with the hypothesized motif in the plasmid (schematically represented at right), and failure of cleavage at those sites was computationally predicted to produce 1.4-kb and 1.9-kb fragments. When EMGBD2_08790 was expressed in the cell, such fragments were actually observed (indicated by red marks). This result supports the hypothesis that the methylation motif of EMGBD2_08790 is TANGG**A**B.

SUPPLEMENTARY TABLES

Supplementary Table 1. Primer sequences employed to clone MTase genes.

Primer name	Sequence (5'→3')
EMGBS3_12600_F	ATCACAAAGTGCATATGATCGCCAGCCGTCCGG
EMGBS3_12600_R	TACCGAGCTCCATATTACTGTTTGCGTAAAATCA
EMGBS15_03820_F	ATCACAAAGTGCATATGAAAAAACAACATAAGGTG
EMGBS15_03820_R	GTACCGAGCTCCATATTATTTATCGCTGCTTGTG
EMGBS10_10070_F	ACCGAGCTCCATAGACTCGTTAAATACTATATTTA
EMGBS10_10070_R	CCGAGCTCCATAGACTCGTTAAATACTATATTTAC
EMGBD2_08790_F	ATCACAAAGTGCATATGAGTAGCACAAGTGTGCA
EMGBD2_08790_R	GTACCGAGCTCCATATTATTTATCACCACCCACTG

F: forward primer; R: reverse primer.

Supplementary Table 2. Accession numbers of the sequence data from this study.

Data set	Sample name	Accession number	Database
Shotgun sequences	biwa_5m	DRX114265-114266	DDBJ Sequence Read Archive
	biwa_65m	DRX114267-114268	DDBJ Sequence Read Archive
Draft genomes	BS1	BGOD01000001-BGOD01000021	DDBJ/ENA/GenBank
	BS2	BGOE01000001-BGOE01000013	DDBJ/ENA/GenBank
	BS3	BGOF01000001-BGOF01000036	DDBJ/ENA/GenBank
	BS4	BGOG01000001-BGOG01000040	DDBJ/ENA/GenBank
	BS5	BGOH01000001-BGOH01000008	DDBJ/ENA/GenBank
	BS6	BGOI01000001-BGOI01000037	DDBJ/ENA/GenBank
	BS7	BGOJ01000001-BGOJ01000006	DDBJ/ENA/GenBank
	BS8	BGOK01000001-BGOK01000034	DDBJ/ENA/GenBank
	BS9	BGOL01000001-BGOL01000003	DDBJ/ENA/GenBank
	BS10	BGOM01000001-BGOM01000024	DDBJ/ENA/GenBank
	BS11	BGON01000001-BGON01000003	DDBJ/ENA/GenBank
	BS12	BGOO01000001-BGOO01000010	DDBJ/ENA/GenBank
	BS13	BGOP01000001-BGOP01000005	DDBJ/ENA/GenBank
	BS14	BGOQ01000001-BGOQ01000006	DDBJ/ENA/GenBank
	BS15	BGOR01000001-BGOR01000044	DDBJ/ENA/GenBank
	BD1	BGOS01000001-BGOS01000030	DDBJ/ENA/GenBank
	BD2	BGOT01000001-BGOT01000011	DDBJ/ENA/GenBank
	BD3	BGOU01000001-BGOU01000010	DDBJ/ENA/GenBank
	BD4	BGOV01000001-BGOV01000049	DDBJ/ENA/GenBank
MTase transfected *E.coli*	EMGBS10_10070	DRX133246	DDBJ Sequence Read Archive
	EMGBD2_08790	DRX133247-DRX133248	DDBJ Sequence Read Archive

Supplementary Table 3. Statistics of metagenomic assembly and genome binning.

Sample	Total length (bp)	Contigs	Longest length (bp)	Average length (bp)	N50 (bp)	Genome bins
biwa_5m	22,609,702	554	481,299	40,812	83,238	15
biwa_65m	10,687,383	345	739,933	30,978	75,701	4

Supplementary Table 4. Methylation motifs detected in the chromosomal DNA of *E. coli* transformed with artificially synthesized MTase genes.

Transformed MTase	Detected methylated motif	Modification Type	Number of methylated sites	Number of motif sequences	Methylation ratio (%)	Mean modification QV	Mean subread coverage
EMGBS10_10070	ACG**A**G	m6A	3,184	3,807	83.6%	48.4	38.2
EMGBD2_08790	TAHGG**A**B	m6A	1,438	2,664	54.0%	60.7	138.0

R= G/A, Y= T/C, M= A/C, K= G/T, S= G/C, W= A/T, H= A/C/T, B= G/T/C, V= G/C/A, D= G/A/T, N= G/A/T/C
