# Supplementary material for: Metaepigenomic analysis reveals the unexplored diversity of DNA methylation in an environmental prokaryotic community
Source: Nat Commun. 2019 Jan 11;10:159. doi: 10.1038/s41467-018-08103-y (PMC6329791; doi:10.1038/s41467-018-08103-y)
Supplement: Supplementary file 3 — Description of Additional Supplementary Files [file 41467_2018_8103_MOESM3_ESM.pdf]

Description of Additional Supplementary Files

File Name: Supplementary Data 1
Description: DNA sequences used for MTase expression.
